# Heterologous expression of formate dehydrogenase enables photoformatotrophy in the emerging model microalga, *Picochlorum renovo*

**DOI:** 10.3389/fbioe.2023.1162745

**Published:** 2023-08-29

**Authors:** Lukas R. Dahlin, Alex W. Meyers, Skylar W. Stefani, Ellsbeth G. Webb, Benton Wachter, Venkataramanan Subramanian, Michael T. Guarnieri

**Affiliations:** ^1^ Biosciences Center, National Renewable Energy Laboratory, Golden, CO, United States; ^2^ Department of Chemical and Biological Engineering, Colorado School of Mines, Golden, CO, United States; ^3^ Renewable and Sustainable Energy Institute, University of Colorado, Boulder, CO, United States

**Keywords:** formate bio-economy, formate, formate dehydrogenase, phototroph, microalgae, picochlorum, photoformatotrophy

## Abstract

Rising global greenhouse gas emissions and the impacts of resultant climate change necessitate development and deployment of carbon capture and conversion technologies. Amongst the myriad of bio-based conversion approaches under evaluation, a formate bio-economy has recently been proposed, wherein CO_2_-derived formate serves as a substrate for concurrent carbon and energy delivery to microbial systems. To date, this approach has been explored in chemolithotrophic and heterotrophic organisms via native or engineered formatotrophy. However, utilization of this concept in phototrophic organisms has yet to be reported. Herein, we have taken the first steps to establish formate utilization in *Picochlorum renovo*, a recently characterized eukaryotic microalga with facile genetic tools and promising applied biotechnology traits. Plastidial heterologous expression of a formate dehydrogenase (FDH) enabled *P. renovo* growth on formate as a carbon and energy source. Further, FDH expression enhanced cultivation capacity on ambient CO_2_, underscoring the potential for bypass of conventional CO_2_ capture and concentration limitations. This work establishes a photoformatotrophic cultivation regime that leverages light energy-driven formate utilization. The resultant photosynthetic formate platform has widespread implications for applied phototrophic cultivation systems and the bio-economy at large.

## Highlights

• Formate is a potential next-generation renewable carbon source for phototroph cultivation.
• Heterologous expression of formate dehydrogenase decreases formate toxicity in *P. renovo.*
• Heterologous expression of formate dehydrogenase enables formate utilization as a carbon source in *P. renovo.*
• Formate supplementation enhances growth under ambient CO_2_ cultivation in formate dehydrogenase expressing strains.

## 1 Introduction

Development of novel CO_2_ sequestration and valorization strategies are urgently needed to reduce greenhouse gas emissions and ameliorate the negative environmental and social impacts of climate change (Foote, 1856; IPCC Pachauri and Meyers, 2014). Indeed, such approaches also present an opportunity to address rapidly increasing global energy and food security demands. To this end, bio-based technologies to convert CO_2_ to fuels, chemicals, materials, and food are actively being evaluated (Grim et al., 2020). Harnessing the power of microbial metabolism to capture and convert CO_2_ represents a high-potential route to enable such bio-based approaches (Barteau et al., 2018). However, microbial cultivation using CO_2_ as a carbon substrate faces a series of challenges, ranging from point source distribution limitations to gas-liquid mass transfer hurdles, and high cellular energy requirements for efficient biological reduction and CO_2_ assimilation (Bar-Even, 2018; Cotton et al., 2020; Grim et al., 2020).

To bypass the hurdles associated with CO_2_ bioconversion, the concept of a formate bio-economy has recently been proposed, wherein CO_2_-derived formate is converted to the aforementioned commodities by leveraging formatotrophic microbial metabolism (Yishai et al., 2016). In one envisioned embodiment, a formate bio-economy would entail the use of renewable electricity to capture and electrochemically reduce either atmospheric (via direct air capture) or point source CO_2_ emissions to formate. This formate could then be upgraded via a variety of formatotrophic microbes to produce sustainable bioproducts (Yishai et al., 2016). This approach presents an opportunity to utilize renewable electricity, while sequestering and converting CO_2_ to formate, thereby directly reducing greenhouse gas emissions.

To date, formate bioconversion has primarily been evaluated in chemolithotrophic and heterotrophic organisms such as *Cupriavidus necator*, *Escherichia coli*, or *Saccharomyces cerevisiae*, via either native formatotrophy or engineered formatotrophic pathways (Wang et al., 2017; Yishai et al., 2018; Gleizer et al., 2019; Gonzalez De La Cruz et al., 2019; Claassens et al., 2020; Kim et al., 2020). For example, microbial formatotrophy has been achieved through FDH-mediated Calvin-Benson-Bassham (CBB) cycle-driven CO_2_ fixation that is native in *C. necator*, or engineered into *E. coli* (Li et al., 2012; Gleizer et al., 2019). Alternatively, higher metabolic efficiency can be achieved via direct formate assimilation pathways (e.g., the reductive glycine pathway) (Gonzalez De La Cruz et al., 2019; Claassens et al., 2020). However, these biological systems require additional reducing power and ATP needed to fix the carbon contained in formate, beyond what can be obtained from formate oxidation itself, ultimately leading to incomplete and/or low-yield carbon fixation and resultant CO_2_ evolution (Yishai et al., 2016). Alternatively, additional sources of reductant can be supplied during cultivation on formate (e.g., hydrogen) to enable improved carbon utilization.

**FIGURE 1:**
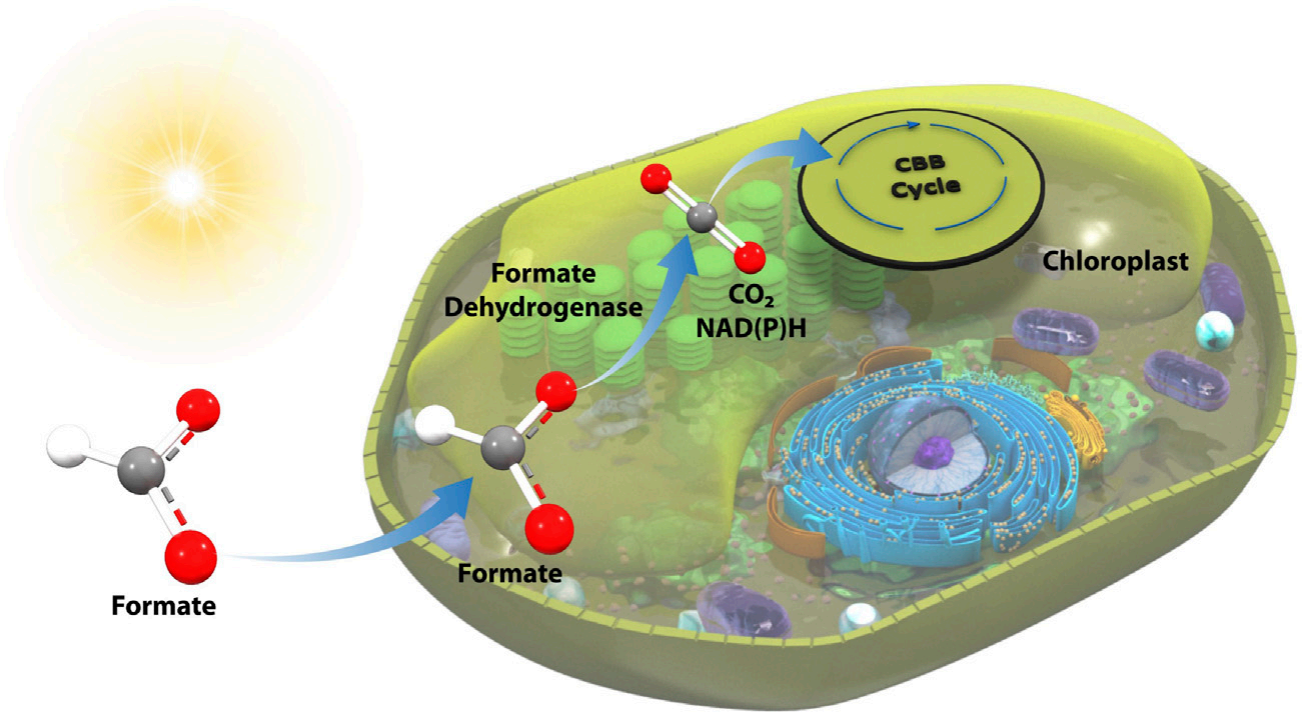
Overview of FDH-mediated photoformatotrophy in *P. renovo*. FDH is transgenically expressed in the *P. renovo* chloroplast, enabling conversion of formate to a reducing equivalent and CO_2_, which can then be assimilated via native metabolism.

Phototrophic organisms present an intriguing, high-potential route to leverage the power of light energy coupled to formatotrophy to enhance growth and enable high carbon utilization efficiency. However, to date, photosynthesis-coupled formatotrophy has yet to be established (Bar-Even, 2018). Herein, we have taken the first steps towards enabling the direct feed of formate as a sole or co-fed carbon and energy source to a phototrophic organism via the integration of a formate dehydrogenase (FDH) into the chloroplast genome of the industrially-relevant microalga, *Picochlorum renovo* (Dahlin et al., 2019; Dahlin and Guarnieri, 2021; Dahlin and Guarnieri, 2022) (Figure 1). The resultant strain is capable of utilizing formate as a carbon and energy source and displays enhanced growth on ambient (0.04%) CO_2_ when supplemented with formate.

## 2 Results

### 2.1 Formate toxicity screening

**FIGURE 2:**
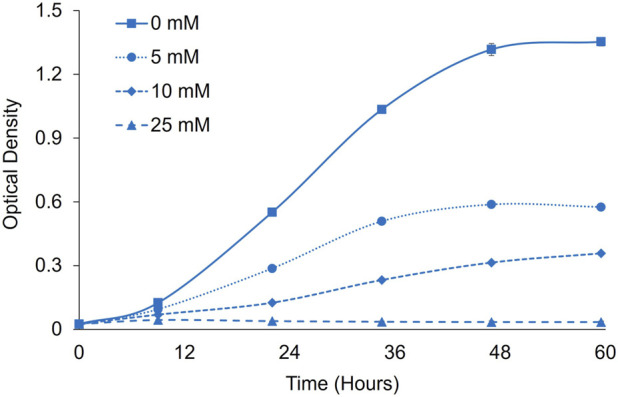
Formate toxicity screening in *P. renovo*. Growth curves of *P. renovo* with varying sodium formate concentrations, at pH = 6.0 in the presence of 2% CO_2_. Data represents the average and standard deviation of 3 biological replicates.

To evaluate formate toxicity and potential for formate utilization in *P. renovo*, we evaluated growth in the presence of 2% CO_2_ with sodium formate supplementation at various concentrations over 60 h (Figure 2). Conventionally, *P. renovo* is cultured at a pH of 7-8 and displays poor growth at pH values < 6 (Dahlin et al., 2019). However, studies in other organisms have shown that low pH (<7) leads to increased formate transport, either through active transport or enhanced passive diffusion of protonated formic acid (Chu et al., 1987; Casal et al., 2008; Wiechert and Beitz, 2017a; Wiechert and Beitz, 2017b; Helmstetter et al., 2019). As such, growth was evaluated at pH 6 via Bis-tris buffering. At this pH, concentrations of 5 mM and 10 mM formate reduced *P. renovo* growth, while a concentration of 25 mM completely inhibited growth (Figure 2). As previously reported, this toxicity is likely due to formic acid transport into the cell and resultant acidification of the cytoplasm upon dissociation to formate and hydrogen ions (Li et al., 2012).

### 2.2 Heterologous formate dehydrogenase expression

**FIGURE 3:**
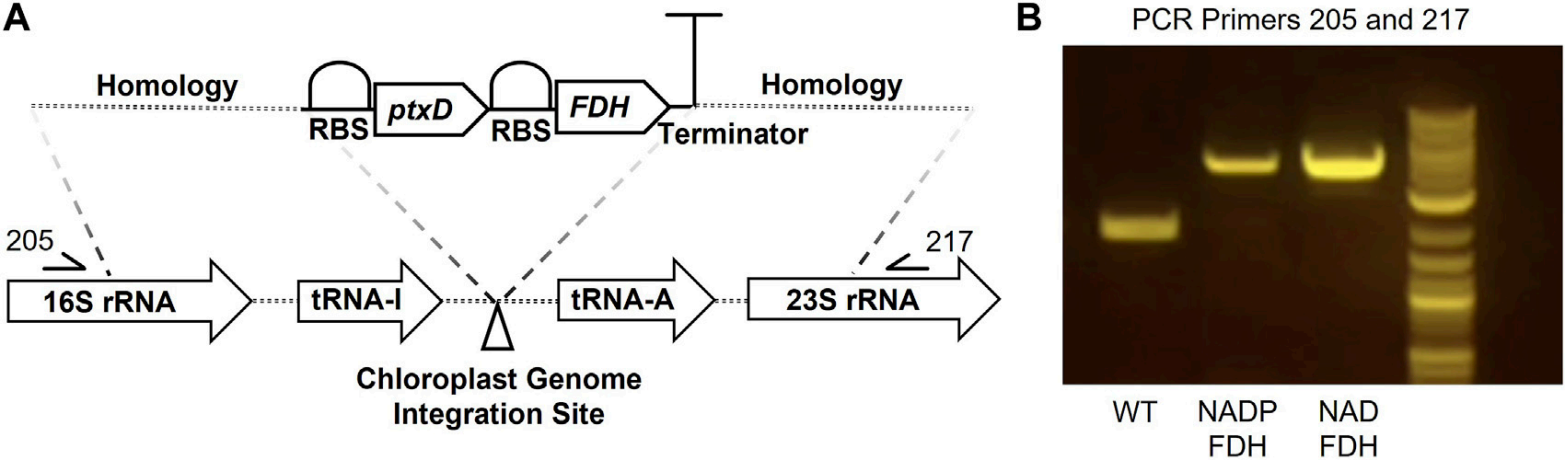
Formate dehydrogenase plastid integration in *P. renovo*. **(A)** Genetic construct for expression of FDH in the chloroplast genome. **(B)** PCR amplification of the formate dehydrogenase insert, verifying homoplasmy of the chloroplast genome. WT, wild-type *P. renovo*; NADP-FDH, transformant *P. renovo* expressing the NADP^+^-FDH variant; NAD-FDH, transformant *P. renovo* expressing the NAD^+^-FDH variant.

To reduce formate toxicity and enable formate utilization, we sought to establish a mechanism by which formate-derived carbon could be assimilated into *P. renovo* CBB metabolism via expression of a FDH (Figure 1). NADP(H) is widely considered the primary nicotinamide cofactor in the chloroplast. However, under active photosynthesis, NADP(H) pools may have limited bioavailability for ancillary reactions and/or may primarily be in a reduced state that would limit FDH activity (Cutolo et al., 2020). To determine if there was a preferred cofactor for the oxidation of formate to CO_2_ in the chloroplast, one FDH mutant that utilizes NAD^+^ and one FDH mutant with preference for NADP^+^ were evaluated (Calzadiaz-Ramirez et al., 2020). These two FDHs were codon optimized to the *P. renovo* chloroplast genome and assembled into our previously established chloroplast integration vector for constitutive expression utilizing phosphite dehydrogenase (*ptxD*) as a selectable marker (Dahlin and Guarnieri, 2022). Transformant algae were obtained via biolistics, and homoplasmy of the chloroplast genomes was confirmed via PCR and Sanger sequencing utilizing primers flanking the insertion site, as described previously (Dahlin et al., 2019; Dahlin and Guarnieri, 2022) (Figure 3).

### 2.3 Formate utilization under high CO_2_ cultivation

**FIGURE 4:**
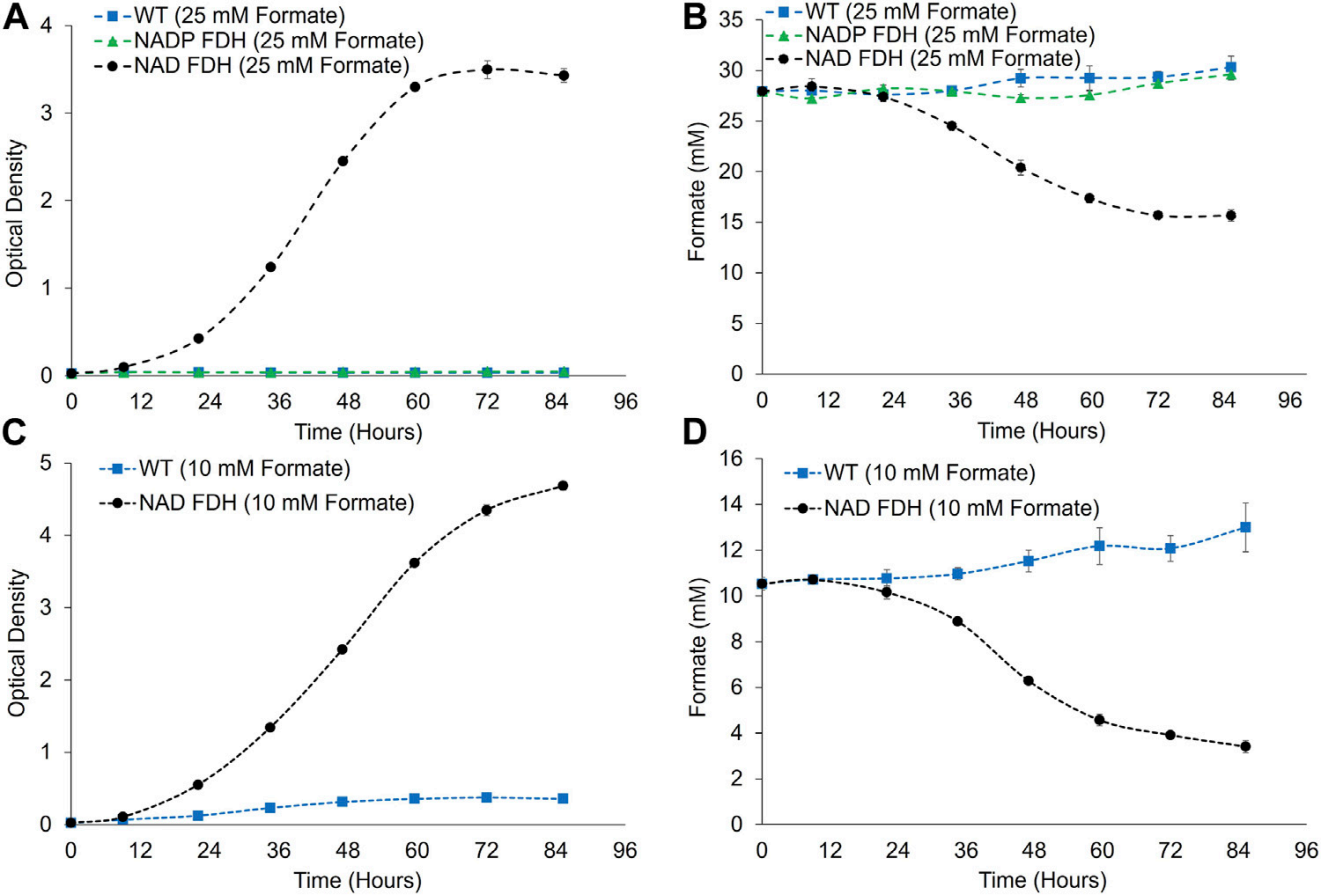
Growth and formate utilization analyses for Wild-type and FDH expressing *P. renovo* supplemented with 25 **(A,B)** and 10 **(C,D)** mM formate. **(A,C)** Growth curves of wild-type, and FDH-expressing *P. renovo* with 25- and 10- mM sodium formate addition at non-growth-limiting (2%) CO_2_ conditions at pH = 6.0. **(B,D)** HPLC analysis of culture supernatant for formate utilization. Data represents the average and standard deviation of 3 biological replicates.

We next evaluated the potential for formate utilization in FDH-expressing strains at non-growth-limiting (2%) CO_2_ concentrations (Figure 4). Growth in media supplemented with 25 mM formate was observed for the NAD^+^-utilizing FDH variant, with 48% ± 1% of formate consumed from the culture media after 85 h of cultivation. Conversely, no growth and no formate utilization were observed for the strain expressing the NADP^+^-utilizing FDH variant. The wild-type culture did not grow on formate and no formate utilization was observed (Figure 4). Following down selection to the NAD^+^-utilizing FDH variant, cultivation capacity on 10 mM sodium formate was assessed to determine if reducing formate levels could decrease residual inhibitory effects of formate and lead to increased growth and percentage of formate utilized. Indeed, a higher culture density was reached when cultivated under 10 mM formate compared to 25 mM formate, potentially due to decreased inhibitory effects when cultivated at lower formate concentrations (Figure 4). Taking evaporative losses into account, formate consumption of the NAD^+^ FDH strain at 85 h was 77% ± 2%. Notably, formate utilization was coincident with growth, with most of the formate consumption occurring during the active growth phase of *P. renovo* (hours 24–72) (Figure 4). Additionally, formate utilization rates were higher when cultivated under 25 mM formate as compared to 10 mM formate, with observed rates of 0.144 ± 0.004 and 0.083 ± 0.006 mmol/h, respectively.

### 2.4 Formate utilization under ambient CO_2_ cultivation

**FIGURE 5:**
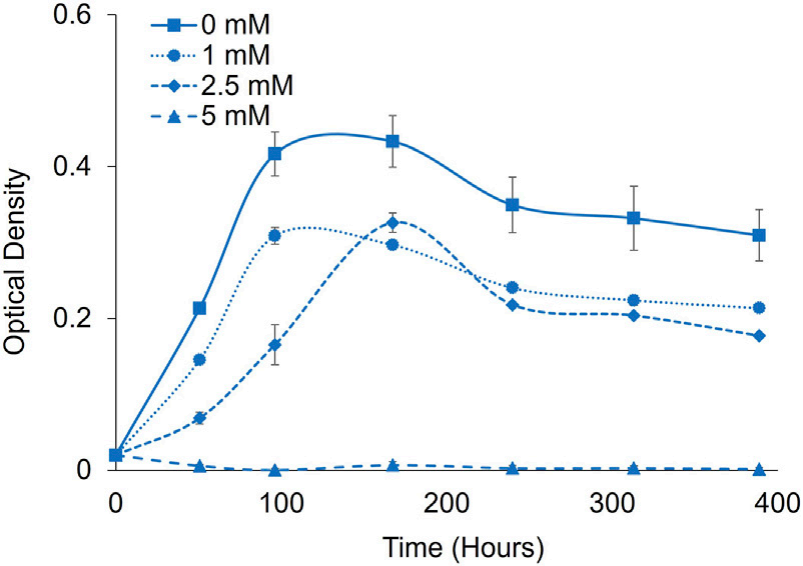
Formate toxicity screening in *P. renovo* at ambient CO_2_. Growth curves of *P. renovo* with varying sodium formate concentrations, at pH = 6.0. Data represents the average and standard deviation of 3 biological replicates.

*P. renovo* grows significantly slower when cultivated on air, compared to 2% CO_2_. To determine the differential impacts on formate toxicity at ambient CO_2_, additional toxicity analyses were conducted at 1-, 2.5- and 5-mM formate at a pH of 6.0 (Figure 5). Under these conditions, 5 mM sodium formate completely inhibited growth.

**FIGURE 6:**
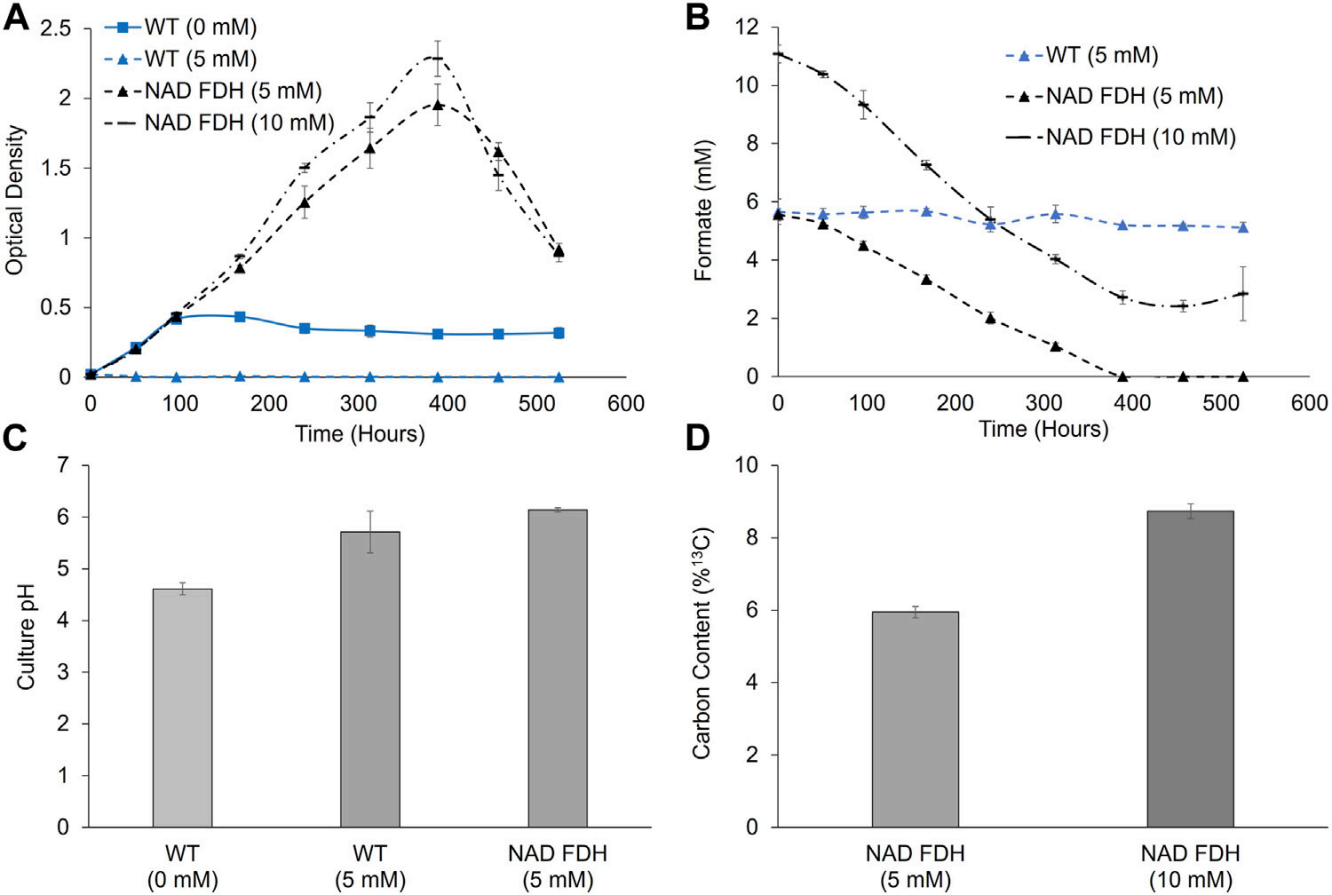
Growth, formate utilization, pH analyses, and ^13^C carbon content for wild-type and FDH expressing *P. renovo* supplemented with varying sodium formate amounts at ambient CO_2_ levels. **(A)** Growth curves of wild-type, and NAD^+^ FDH-expressing *P. renovo* with varying sodium formate additions at growth-limiting ambient (0.04%) CO_2_ conditions at pH = 6.0 **(B)** HPLC analysis of culture supernatant for formate utilization. **(C)** pH values after 380 h of cultivation for 0- and 5-mM formate cultures. **(D)** Endpoint ^13^C analysis of biomass, reporting the percent of ^13^C when grown on labeled sodium formate. Data represents the average and standard deviation of 3 biological replicates.

Following ambient CO_2_ toxicity screening, we next analyzed growth at ambient concentrations of CO_2_ to determine if exogenously supplemented formate (using ^13^C sodium formate) could lead to a growth enhancement and incorporation of carbon derived from formate into the biomass under CO_2_-limited conditions. As shown in Figure 6, cells expressing FDH displayed enhanced growth when supplemented with 5- or 10-mM formate, growing to a higher final culture density than those without formate supplementation. Formate concentrations dropped from the initial starting concentrations, with complete utilization for the 5 mM culture and 78% ± 1% for 10 mM, as measured via HPLC. Under these same conditions, no formate utilization was observed in wild-type cultures (Figure 6). The initial growth rates of wild-type and NAD^+^-FDH-expressing strains were equivalent. However, following ∼100 h of cultivation, the unsupplemented wild-type culture enters stationary phase whereas the supplemented NAD^+^-FDH-expressing strain continues to grow to > 4.3X optical density relative to wild-type. Increasing the formate concentration from 5 mM to 10 mM led to an increase in final optical density of 2.0–2.3. Similar to the results observed at 2% CO_2_, formate utilization rates were higher when cultivated under 10 mM formate compared to 5 mM, with rates of 0.022 ± 0.001 and 0.014 ± 0.001 mmols/h, respectively. To highlight changes in media pH due to consumption of ammonium chloride (lowering pH) and sodium formate (increasing pH), we analyzed pH at hour 380. Notably, wild-type cultures without added formate decreased in pH, while the FDH expressing strain with 5 mM formate maintained the initial starting pH. Finally, ^13^C analysis confirmed incorporation of the carbon contained in the formate into cellular biomass, with 6.0% and 8.6% ^13^C for 5 and 10 mM, respectively, compared to the natural abundance of ∼1.1%.

Given that low pH cultivation is suboptimal for *P. renovo* growth, we also assayed cultivation capacity of wild-type and the NAD^+^ FDH expressing strain on 0- and 5- mM formate at a pH of 7 (Supplementary Figures S1, S2). In regard to formate toxicity, supplementation with 5 mM formate led to equivalent growth as 0 mM, i.e., 5 mM was not toxic in the wild-type strain. In the absence of formate supplementation, increased growth in the wild-type culture was observed, reaching a peak OD of 1.6, compared to 0.4 for pH 6.0. However, the NAD^+^ FDH expressing strain had lower formate utilization at pH 7.0, with 63% ± 5% utilized after 525 h of cultivation. This represents a rate of 0.007 ± 0.001 mmol/h, lower than that observed above for cultivation at the lower pH of 6.0. Decreased formate utilization capacity at pH 7.0 was further corroborated by ^13^C analysis, wherein only 4% of the biomass was labeled, compared to 6% for the equivalent culture at a pH of 6.0.

## 3 Discussion

CO_2_ delivery has been predicted to account for nearly 20% of algal biomass production costs and also presents carbon utilization efficiency (CUE) hurdles due to poor gas-liquid mass transfer and rapid off gassing in open systems (Davis et al., 2016). Improved carbon delivery and CUE could be achieved via the direct feeding of water-soluble formate to phototrophic systems, which would concurrently deliver necessary carbon and reducing equivalents for growth. Additionally, the relatively low concentration of atmospheric CO_2_ can be a key limiting factor in terrestrial phototroph productivity. Therefore, photoformatotrophy could also be deployed in terrestrial crops to enhance productivity in support of a bioeconomy and increasing global food production demands (Blankenship et al., 2011; Hann et al., 2022).

To fully bring to bear the potential of photoformatotrophy, a series of key conversion hurdles will require targeted bypass. Enhancement of formate utilization may be achieved by targeting a number of interacting variables, including formate/formic acid transport rate across the cell membrane, which may occur via passive or active transport mechanisms. Additionally, the activity of the expressed FDH may be limiting and presents a high-potential target for protein engineering and screening. Finally, the pool of intracellular oxidizing equivalents in the form of NAD(P)^+^ can be targeted.

With regard to formate transport, genomic analysis of *P. renovo* identified a putative formate/nitrite transporter with 39% homology to the *fdhC* formate transporter in *Methanobacterium formicium*. This *fdhC* homolog also encodes a conserved formate/nitrite transporter domain with 6 associated transmembrane domains, which could be responsible for formate transport in this alga, in conjunction with passive diffusion (White and Ferry, 1992; Wood et al., 2003; Hallgren et al., in press). Genetic engineering and culture optimization for increased formate transport is an area of future work that could be achieved through heterologous expression of various characterized formate transporters, or through manipulation of culture pH to concurrently optimize formate transport and cellular growth (White and Ferry, 1992; Wang et al., 2009; Wiechert et al., 2017). It is also important to note that with the cultivation strategy proposed herein, formate needs to be transported across both the cell and chloroplast membranes, potentially necessitating transporters for both membrane localizations.

Notably, under the conditions tested here, as *P. renovo* utilizes sodium formate an OH^−^ anion is generated, which can increase culture pH. Conversely, consumption of ammonium salts (such as the ammonium chloride utilized herein) will decrease culture pH via formation of a H^+^ ion (Calvey et al., 2023). This can lead to alterations in pH that respectively decrease formate transport or inhibit growth. These phenomena explain the observed pH differences in Figure 6 and large growth differential between wild-type cultures without formate compared to NAD^+^ FDH expressing cultures supplemented with formate, despite the relatively low (∼6–8%) ^13^C labeling. The increase in growth with formate supplementation is thus likely due to a combination of increased carbon availability from formate coupled to a more favorable growth pH induced by formate oxidation. Future work will eliminate these pH fluctuations by the addition of ammonium hydroxide (as a cellular nitrogen source) and formic acid in pH-stat fed bioreactors, which results in no net change to culture pH as formic acid and ammonium hydroxide are utilized (Calvey et al., 2023). Additionally, given the incongruence between optimal growth and formate utilization pH, future work will need to target enhancement of transport at neutral pH (e.g., via transporter engineering) or adaptation of *P. renovo* for improved growth at acidic pH (e.g., via adaptive laboratory evolution).

Alternatively, inherent FDH kinetics and cofactor specificity may limit FDH activity, and thus hinder formate utilization. As such, screening of alternative FDH variants offers a promising route to identify FDH with increased formate utilization kinetics. At a high level, known FDH enzymes are separated into two classes, metal-independent, and metal-dependent. While the metal-independent class is generally less cumbersome for heterologous expression, due to single subunit functionality (such as the *Pseudomonas* variant utilized herein), metal-dependent FDHs are generally more complex and have more favorable kinetics (Il Oh and Bowien, 1998; Moon et al., 2020; Young et al., 2020). Localization of the FDH offers a further opportunity for optimization; for example, addition of a RuBisCO binding motif to the FDH may localize the FDH to RuBisCO, such that CO_2_ produced from formate oxidation is readily available for fixation by the enzyme (Itakura et al., 2019; Meyer et al., 2020). In the results presented herein, the NADP^+^ utilizing FDH variant did not grow in the presence of formate, suggesting minimal to no functionality. This was unexpected, as NADP^+^ is generally considered to be the most abundant dinucleotide cofactor in the chloroplast (Cutolo et al., 2020). The lack of NADP^+^ FDH functionality in *P. renovo* could be due to a higher proportion of NADPH, limiting the availability of non-reduced NADP^+^ equivalents needed for FDH functionality, or the relatively poor enzyme kinetics of the NADP^+^-utilizing FDH variant (Calzadiaz-Ramirez et al., 2020).

Finally, NAD^+^ levels may limit formate utilization by failing to provide sufficient oxidizing equivalents needed for FDH activity. NAD^+^ levels in phototrophic systems may be increased through either limiting light intensity or decreasing light absorption by the photosynthetic antenna. However, such approaches could limit photo-productivity. Alternatively, metabolic pathways that require large amounts of reducing equivalents could be upregulated, or novel pathways introduced, such as starch, lipid, or terpenoid biosynthesis, which would in turn produce useful biochemical intermediates while regenerating needed oxidizing equivalents for formate utilization.

In summary, we have taken the first steps towards engineering a phototroph for formatotrophy, establishing proof-of-concept for photoformatotrophy. This strategy offers the potential for a series of benefits to enhance the productivity of phototrophs via the delivery of reduced carbon in the form of formate that can be readily produced from CO_2_ via electrolysis. First, in comparison to gaseous substrates such as CO_2_, formate is notably easier to both store and transport (Cotton et al., 2020). Second, formate is completely miscible in water thereby increasing mass transfer while decreasing potential for CO_2_ off gassing which ultimately manifests as low system CUE. Third, formate also enables the ultimate conversion of electrical energy to cellular energy (i.e., reducing equivalents), in turn enabling higher cell density cultivation. Fourth, formate is broadly toxic to many organisms, as such, contamination can be greatly reduced, which can lead to drastic declines in biomass yields during cultivation of both aquatic and terrestrial phototrophs (Grunwald et al., 2015; Claassens et al., 2020; Cotton et al., 2020). While a number of these benefits apply to aquatic species, application of formate feeding to higher plants represents an additional exciting area of future work. Finally, this work lays the foundation for incorporation of more efficient, direct formate utilizing pathways, such as the reductive glycine and formolase pathways, and integration with microbial electrosynthesis approaches wherein formate serves as an electron and carbon mediator molecule, to ultimately enable a photosynthetically-driven formate bio-economy (Bar-Even et al., 2013; Bar-Even, 2016; Bar-Even, 2018; Claassens et al., 2019; Cotton et al., 2020; Naduthodi et al., 2021).

## 4 Methods

### 4.1 Strain and cultivation conditions

Formate toxicity screening was carried out utilizing a modification of our previously described media (Dahlin et al., 2019). Media was prepared with 250 mL of seawater (Gulf of Maine, Bigelow Labs), and 750 mL of deionized water. Macro nutrient concentration was 5 mM N (as NH_4_Cl), and 0.313 mM P (as NaH_2_PO_4_). Trace metals were 1.06 × 10^−4^ M Si (as Na_2_SiO_3_9H_2_O), 1.17 × 10^−5^ M Fe (as FeCl_3_ 6H_2_O), 1.17 × 10^−5^ M EDTA (as Na_2_EDTA 2H_2_O), 3.93 × 10^−8^ M Cu (as CuSO_4_ 5H_2_O), 2.60 × 10^−8^ M (as Na_2_MoO_4_ 2H_2_O), 7.65 × 10^−8^ M Zn (as ZnSO_4_ 7H_2_O), 4.20 × 10^−8^ M Co (as CoCl_2_ 6H_2_O) and 9.10 × 10^−7^ M Mn (as MnCl_2_ 4H_2_O). Vitamins were added as follows, thiamine HCl (2.96 × 10^−7^ M), biotin (2.05 × 10^−9^ M) and cyanocobalamin (3.69 × 10^−10^ M). Trace metal, silica and vitamin stock solutions were purchased from Bigelow Labs. Media was buffered with 10 mM Bis-Tris, and media pH was adjusted to 6.0 using concentrated HCl.

Sodium formate (HCO_2_Na) was added to the above media to obtain the desired formate concentration for experiments at 2% CO_2_, ^13^C sodium formate (Sigma 279412) was utilized for experiments done at ambient CO_2_. To assay for formate toxicity, 45 mL of culture (in a 250 mL Erlenmeyer flask) was inoculated from mid log phase cells to an optical density (750 nm) of 0.025. Cultures were mixed via shaking (170 rpm) at 33°C, 2% CO_2_, and 125 uE cool white LED lighting. For experiments relating to formate utilization, the above conditions and media were used, with varying CO_2_ concentrations in a Percival Scientific growth chamber.

### 4.2 Construct assembly and transformation

FDH variants utilized were mutated from the *Pseudomonas* sp. 101 FDH, specifically NAD^+^ utilizing variant (A198G) and NADP^+^ utilizing variant (A198G/D221Q/C255A/H379K/S380V), as described in Calzadiaz-Ramirez et al. (2020). FDH transformation vectors were prepared by Twist Bioscience, cloning a ribosomal binding site (AGGAGGTTATAAAAA) and codon optimized (Geneious Prime, Supplementary Table S1) FDH downstream of the *ptxD* selectable marker in our previously described chloroplast transformation vector (Dahlin and Guarnieri, 2022). *P. renovo* transformation was carried out as described previously, with the exception that Critter Technology binding and precipitation buffers were used according to the manufacturers recommendations to bind DNA (plasmid prepared by Twist Bioscience) onto the gold microcarriers for biolistic transformation (Dahlin et al., 2019; Dahlin and Guarnieri, 2022).

### 4.3 Formate quantitative analysis

Formate quantification was carried out by high performance liquid chromatography using an Agilent 1,100 series system. Six µL of filtered cell-free supernatant was used for injection into the Bio-Rad HPX-87H (300 × 7.8 mm) ion exchange column. Elution of the organic acid was carried out with 0.01 N sulfuric acid at a flow rate of 0.6 mL per min. The column temperature was maintained at 55°C. The retention peak time was recorded using Chemstation software followed by quantification using a standard curve generated for formate.

### 4.4 ^13^C analysis

^13^C analysis was performed on freeze dried algal biomass collected at the endpoint of cultivation (hour 525 in Figure 6). Briefly, aliquoted samples were combusted with a Flash 2000 elemental analyzer (Thermo), with isotope abundance measured via an attached continuous-flow stable isotope ratio mass spectrometer (Delta V; Thermo), as described previously (Henard et al., 2021). Sample values were corrected for offset and scale using a 3-point scale correction and a suite of isotope and organic content standards. Calculations and data correction were performed using a suite of R scripts using R statistical software (v4.2.0) and the RStudio 2022.07.0 interface with Tidyverse and IsoVerse packages (Kopf et al., 2021; Wickham et al., 2023).

## Data Availability

The original contributions presented in the study are included in the article/Supplementary Material, further inquiries can be directed to the corresponding authors.
