# Supplementary material for: Heterologous expression of formate dehydrogenase enables photoformatotrophy in the emerging model microalga, Picochlorum renovo
Source: Front Bioeng Biotechnol. 2023 Aug 29;11:1162745. doi: 10.3389/fbioe.2023.1162745 (PMC10497104; doi:10.3389/fbioe.2023.1162745)
Supplement: Supplementary file 2 [file DataSheet1.docx]

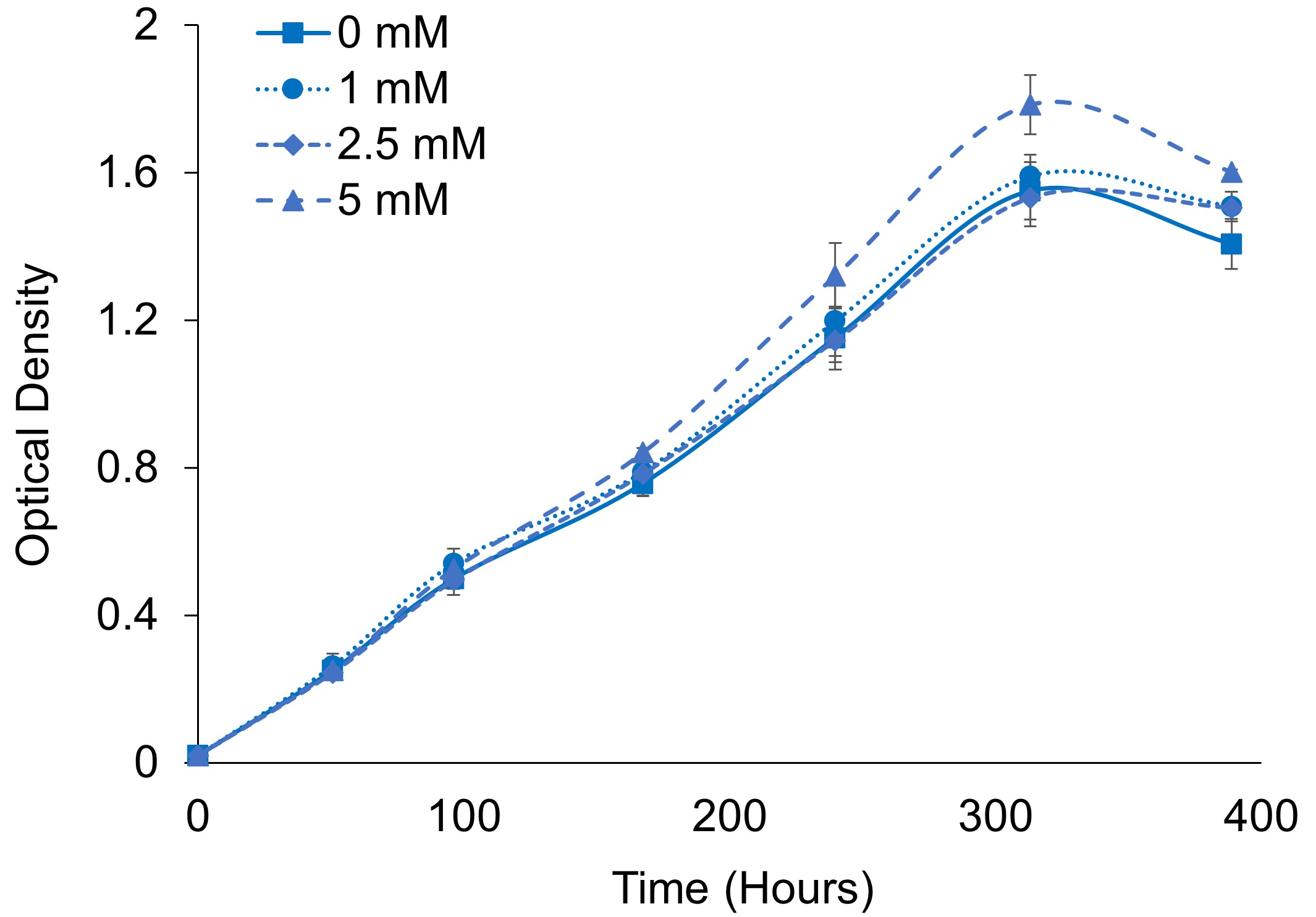


**Supplemental Figure 1. Formate toxicity screening in *P. renovo* at ambient CO_2_.** Growth curves of *P. renovo* with varying sodium formate concentrations, at pH = 7.0. Data represents the average and standard deviation of 3 biological replicates.


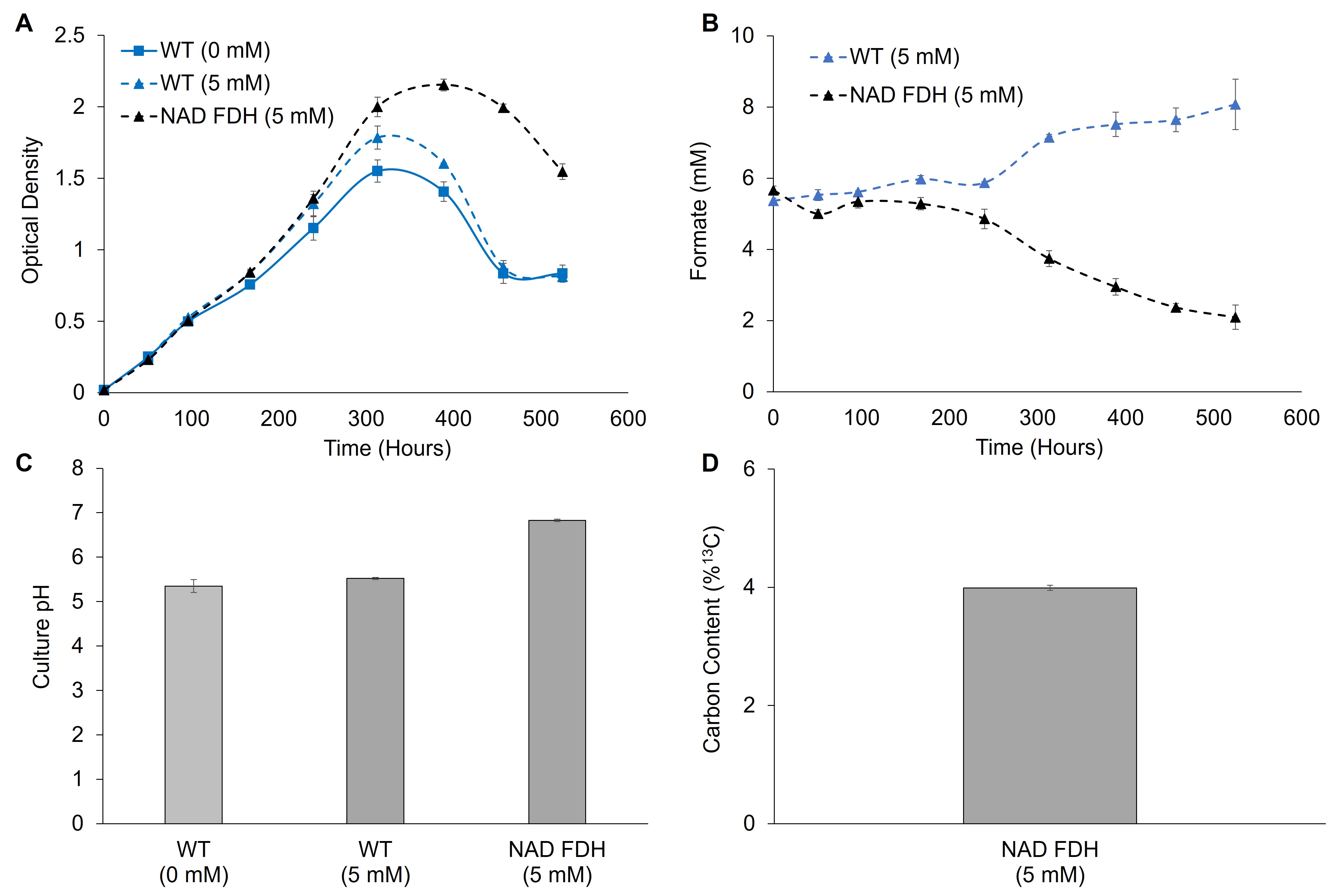


**Supplemental Figure 2. Growth, formate utilization, pH analyses, and ^13^C carbon content for Wild-type and FDH expressing *P. renovo* supplemented with varying sodium formate amounts at ambient CO_2_ levels. (A)** Growth curves of wild-type, and NAD^+^ FDH-expressing *P. renovo* with varying sodium formate additions at growth-limiting ambient (0.04%) CO_2_ conditions at pH = 7.0 **(B)** HPLC analysis of culture supernatant for formate utilization. **(C)** pH values after 380 hours of cultivation. **(D)** Endpoint ^13^C analysis of biomass, reporting the percent of ^13^C when grown on labeled sodium formate. Data represents the average and standard deviation of 3 biological replicates.
